# The discovery, positioning and verification of a set of transcription-associated motifs in vertebrates

**DOI:** 10.1186/gb-2005-6-12-r104

**Published:** 2005-12-02

**Authors:** Laurence Ettwiller, Benedict Paten, Marcel Souren, Felix Loosli, Jochen Wittbrodt, Ewan Birney

**Affiliations:** 1 EBI, Wellcome Trust Genome Campus, Hinxton, Cambridgeshire, CB10 1SD, UK; 2 EMBL, Meyerhofstrasse, 69012 Heidelberg, Germany

## Abstract

We have developed several new methods to investigate transcriptional motifs in vertebrates. We developed a specific alignment tool appropriate for regions involved in transcription control, and exhaustively enumerated all possible 12-mers for involvement in transcription by virtue of their mammalian conservation. We then used deeper comparative analysis across vertebrates to identify the active instances of these motifs. We have shown experimentally in Medaka fish that a subset of these predictions is involved in transcription.

## Abstract 2

Several new methods and a specific alignment tool for investigating transcriptional motifs in vertebrates were used to identify all possible 12mers involved in transcription. Active instances of these motifs were identified using comparative analysis across vertebrates.

## Background

A genome encodes more than just the structural proteins or RNA sequences that form active biological molecules. In addition, the control of expression of these structural genes is determined by elements that act at the DNA, RNA or epigenetic level and are associated with specific genes in some manner. Considerable knowledge of these regulatory networks is available for specific sets of genes; for example, the network of largely transcription based control involved in muscle specific gene expression in mammals [1], or the control of sex determination in the Drosophilids [2], which is primarily via regulation of RNA processing. In the case of transcriptional control, these elements work by modulating the rate of transcription from promoters (reviewed in [3]). Surprisingly, we have no strong computational model to allow us to predict where the genomic elements involved in gene expression lie despite often detailed knowledge of certain control elements, perhaps best illustrated by the set of genes involved in the development of the sea urchin [4]. This is true either in a whole genome context or when one restricts the problem to areas suspected to be involved, for example, regions directly upstream of genes. In contrast, for constitutive RNA processing of pre-mRNA molecules, we have computational models that provide reasonably good predictions, through programs such as Genscan [5] and Fgenesh [6]. Perhaps more importantly, these computational models have allowed the development of programs, such as Genewise [7], Genie [8] and est2genome [9], that integrate experimental data and gene model aspects to provide highly accurate gene prediction. We have not found all the protein coding genes in any large genome, but we do have a good sense of where a large portion of the genes are located due to this computational model. Having a practical, predictive model for the transcriptional elements of a genome would provide a significant advance in the understanding of the regulation of specific genes and the interpretation of mutations that are associated with human disease.

We, like many researchers, make a distinction between short 'motifs' and longer 'regions' involved in cis-regulation. For an excellent review on the subject with a discussion of evolutionary aspects see Wray *et al*. [10] and for a review from the bioinformatics perspective see Wasserman and Sandelin [11]. A motif is a subsequence of DNA of between 6 and 20 base pairs (bp) of fixed or almost fixed width. In most cases, each motif has a particular sequence consensus that generalizes all copies of the motif. It is thought that a single factor or a small multimeric complex of transcription factors binds the motif, and the sequence consensus is a property of this binding. Regions are far longer, up to approximately 1,000 bp of genomic sequence. The promoter can be classed as a region just proximal to the transcription start whereas enhancers or locus control regions are regions some distance from the promoter. This simplistic classification by distance probably incorrectly combines and separates underlying mechanistic classes. Generalizing from the elegant work done on specific examples [4], we expect that most regions have clusters of motifs that somehow act synergistically.

One perplexing aspect of transcriptional control mediated by cis-regulatory motifs is that, in large genomes, one expects and observes between 10e4 to 10e6 instances of each motif in the genome. It is hard to imagine that all these instances are equally likely to be occupied, with transcriptional control occurring via this occupancy. Suggested reasons to reconcile the direct experimental evidence of binding affinities with this large excess of potential sites include epigenetic features, in particular chromatin modeling and methylation, and cooperative binding of complex combinations of motifs that allows multiple weak signals to be combined to provide specificity. For an excellent review of this area see Jenuwein and Allis [12]. Sadly, the epigenetic factors are not as amenable to experimental analysis as the raw DNA sequence, though there has been considerable progress in recent years [13,14]. More importantly for this paper, these aspects are hard to model computationally.

Previous attempts at computational investigations of cis-regulation have focused on three main avenues of attack. One is to build carefully curated results of direct experimental work, in the hope that either there are enough experiments to effectively cover a particular genome or that such collections provide useful computational generalizations applicable to the whole genome. The TransFac database [15] and the Transcription Regulatory Regions Database (TRRD) [16] are good examples of this approach, and in our hands we find the Jaspar database [17] the most accurate representation of known transcription factor binding data. The second approach is to use large scale experimental techniques, in particular chromatin immunoprecipitation followed by large scale assay using microarrays, so called chIP on Chip techniques [18,19]. The final approach is to use pure bioinformatics investigation of genome sequences. Conventionally, researchers have combined genome data with a second dataset. Two datasets are commonly used; gene expression data [20-24] and comparative data such as in [25]. Many groups have had considerable success in studying motifs in *Saccharomyces cerevisiae*, including comparative genomics approaches [26]. In our own previous work, we have used protein-protein interaction data and metabolic information in combination with the yeast genome to provide an effective (although partial) investigation [27]. Comparative information is often used in more limited studies when a researcher is only interested in a small set of genes, using methods commonly termed 'phylogenetic footprinting' [25]. As most of these techniques need several relatively close species to be sequenced to be effective, many of these phylogenetic techniques are not yet applicable genome-wide in vertebrates. The recent paper by Xie *et al*. [28] shows the current state of the art in this area: using four genome sequences they were able to identify motifs that were over-represented in conserved regions around genes, and showed that these motifs are non-randomly distributed with respect to gene expression data. Xie *et al*. were not able, however, to identify the specific instances of the motif that were the active copies of these motifs in the genome. The 'evolutionary selex' method presented in this paper is similar to the Xie *et al*. technique and was developed independently.

In this paper we propose a novel genome-wide computational method that also uses comparative genomics in two distinct stages. Similar to the Xie *et al*. method, we do not attempt to make direction predictions of motif positions on genomes from individual promoter sequences. Instead we aim to predict an accurate dictionary of motifs with statistical properties that seem specific to cis-regulatory motifs using a technique we have called 'evolutionary selex' with inter-mammalian alignments. Specifically for this project, we developed a novel alignment routine that we believe models more closely promoter evolution and show in passing that for most, but not all, cases promoter elements seem to remain co-linear over human/mouse evolutionary distances. We then used an efficient method to allow direct enumeration of all possible motifs up to 12-mers, including motifs with wild cards. This brute force enumeration means that we do not have a machine learning optimization problem to solve. We therefore have independently confirmed the generation of a motif set using comparative genomics, similar to the Xie *et al*. paper, but we extended this work to find specific instances. We used a more distant comparative genomics approach of over-representation in related orthologs across vertebrates to identify specific instances for these motifs. We show by direct experiments in Medaka fish that these active motifs are necessary to drive expression *in vivo *and their removal affects transcription.

## Results

### Alignment of promoters

We wished to develop an alignment program focused on the evolution of regions involved in transcriptional processes. We reasoned that such a tool should be tolerant to inversions and translocations as well as the more usual insertions and deletions. We also felt that long insertions or deletions should be tolerated. When considering inversions or translocations, the resulting alignment grammar becomes a context-sensitive style grammar, and there is, therefore, no polynomial time method to find a maximum score for a given scoring scheme of these events [29]. We therefore used a pragmatic heuristic of seeding from small ungapped alignments followed up by a series of local alignments using the DNA Block Aligner (DBA) alignment model [30] implemented in the program promoterwise (see Materials and methods for more detail).

**Figure 1:**
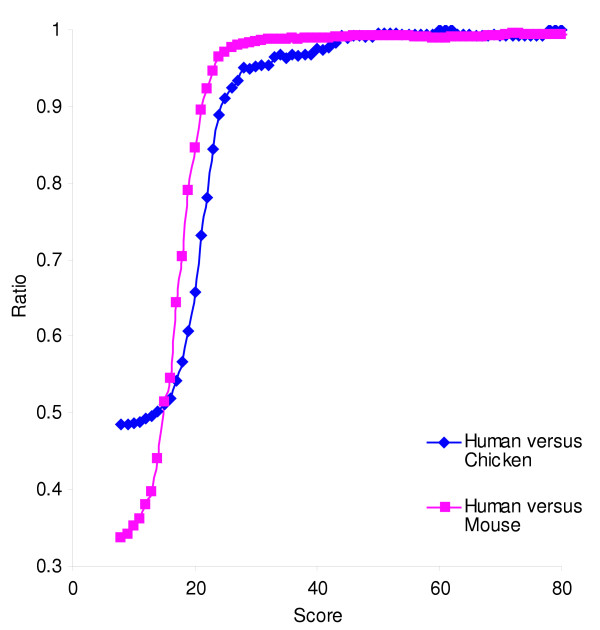
A plot showing the ratio of +/+ orientation promoterwise alignments (being collinear with the direction of transcription) versus all alignments for human/mouse (blue lines) and human/chicken (magenta) promoters. The x-axis is the bit score range, binned in 1 bit intervals. The y-axis is the ratio of +/+ alignments to the total number in this range. All species except mouse/rat show similar 'step' behavior between 20 and 25 bits. The depression below 0.5 of mouse/human alignments at low bit scores at first seems surprising, as one expects random data to show a 0.5 ratio. This depression is because there is a significant amount of +/+ alignments which, when close to random alignments, will often capture low scoring alignments, especially if it is straddled by two high scoring alignments, and merge into one high scoring alignment. As this predominantly occurs in forward/forward alignments, this means that there is a depression of low scoring forward/forward alignments.

The DBA method is parameterized as a probabilistic model of short, relatively gap-free conserved sequences compared to a null model of unrelated bases [30]. The natural scoring method of such a probabilistic model is to report the log of the likelihood ratio of the two models, which is calculated in a single dynamic programming routine. The likelihood ratio could be used to generate a posterior probability assessment of the significance of each alignment, but one would still need to choose a prior probability for the chance of seeing an alignment before examining the data. This prior becomes equivalent to a threshold of log-odd likelihood score above which one believes the alignment to be significant. We investigated a number of properties of both real and random promoterwise alignments select this threshold. We performed simulation studies with random sequence that showed that bit scores >20 bits are extremely rare when aligning randomly generated sequences. Turning to real alignments, we compared promoter regions from several different species pairs, in each case taking orthologous genes from Ensembl and using the 5 kb upstream of the longest transcript to define the potential promoter. As the bit score cutoff was increased, a greater fraction of the alignments matched the direction of transcription in both genes. A striking discontinuity was observed around 20 bits (Figure 1). Other characteristics of promoterwise behavior also changed at around 20 bits, including a sharp discontinuity of the number of pairs of orthologs showing alignment of this score or higher.

We compared promoterwise alignment to other alignment methods, in particular BLASTZ [31], which is a robust and well tested heuristic method based around a Smith-Waterman style alignment. BLASTZ has a scoring scheme tuned to cover the maximal amount of human/mouse orthologous base pairs. Promoterwise alignments greater than 25 bits are found 96% of the time inside BLASTZ alignments but represent only 13% of the BLASTZ aligned base pairs. When the 'tight' scoring matrix used by the University of California Santa Cruz Genome Browser Group (UCSC) is applied to the BLASTZ alignments, only 42% of the promoterwise alignments overlap tight BLASTZ alignments. A similar comparison to LAGAN alignments (from a four-way MLAGAN across human, mouse, dog and rat, and then taking the projected pairwise human/mouse alignment) showed similar results of promoterwise alignments being a specific subset of the LAGAN alignment, but not a different alignment of the bases.

Our interpretation is that the promoterwise scoring scheme with a 25 bit cutoff selects for a particular subset of DNA that is likely to be under negative selection. This is because of the sharp increase of the strand ratio of the alignments towards mainly collinear orientations, suggesting that a different process from random alignments (including neutral inversions or translocations) is occurring. Furthermore we will assume later on that these negatively selected alignments will be enriched in functional sequences in promoters and that these are most likely to be transcriptional motifs. This is because we expect that removal of transcriptional motifs would, in general, be detrimental to the organism. At closer distances (for example, mouse/rat) we observed different behavior, probably due to neutral DNA still aligning because the neutral inversions have not had enough time to accumulate 'drift' mutations. In human, we produced a set of negatively selected DNA from the comparison with mouse in the upstream regions of 10,300 genes, totaling 6,571,106 bp (0.21% of the human genome).

### Motif discovery by evolutionary selex

We wished to use this negatively selected pool of DNA to discover motifs. We investigated several objective functions that could distinguish potential cis-regulatory motifs from other motifs. A poor result was observed when using over-representation of motifs in promoter sequences versus background genome (data not shown). In our hands, an excellent objective function was the relative distribution of motifs in conserved versus non-conserved regions in significant promoterwise based alignments (see Materials and methods). We term this approach 'evolutionary selex' as it mimics the selex method [32] of discovering the binding site of a motif by looking at a population of sequences that satisfy a criterion. Rather than using immunoprecipitation to select these sequences, we used evolution to enrich our sequence pool. There are two main challenges to solve here: finding the right metric to confidently distinguish a real motif from the background and then a way to use this metric to find new motifs.

### Statistics of small subsequences in conserved regions

**Figure 2:**
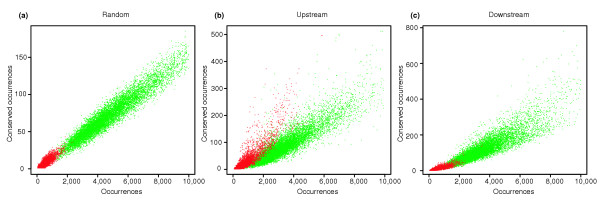
Three panels showing the conservation versus occurrence of all 7-mer words in three different areas of the genome. **(a) **Random regions. **(b) **Regions 5 kb upstream of genes. **(c) **Regions 5 kb downstream of genes. Each word is colored either red if it has one or more CG dinucleotides or green otherwise.

The relationship between the occurrence of motifs in the restricted regions of negative selection versus overall occurrence in promoters can be seen in Figure 2, which shows this ratio for three different regions of the human genome for all 7-mer words. The choice of 7-mers is to show reasonably complex word behavior for this discussion; the enumeration described later tests all n-mers up to 12. Notice that for both randomly chosen and downstream regions there seems to be a well defined relationship between the total occurrence of a motif and its occurrence in these conserved regions. The CG motifs show classic suppression across the genome. The well understood phenomena of cytosine methylation on CpG dinucleotides allows the methylated cytosine to mutate far faster than any other base pair in the genome, leading to a relative lack of CG dinucleotides in the genome except in unmethylated regions.

The downstream and random distributions are reasonably well modeled by a simple binomial distribution where there is some probability of landing in a conserved region, so that, for a given overall occurrence of a motif, a proportion of the motifs randomly fall in these conserved regions. The shape of the distribution is a good fit but there is too much variance of the conserved number for a particular occurrence number. We believe this is simply due to non-random behavior of words in the human genome (probably changing the total occurrence number in a complex manner). Given that the shape of the distribution is a good model, however, we believe that motifs >10 standard deviations can be considered very non-random and thus interesting for further study.

Figure 2 shows the ratio of occurrence versus conservation for upstream regions. This plot is radically different from the other plots: most obviously the CG containing motifs are behaving separately from their non-CG peers. More subtly, there are many more motifs in the top left side of the distributions (found more times in conserved regions than their peers of similar overall occurrence). This radically different behavior indicates that conservation is behaving differently with respect to words in upstream regions. A complex relationship between occurrence and conservation counts, however, prevents a simple statistical model. In particular, there is no single model of the distribution we can use for both the CG containing motifs and non-CG containing motifs. As well as it being unsatisfying to have to separate these cases, this dual distribution precludes us from combining non-CG and CG motifs sensibly when wild cards are used.

**Figure 3:**
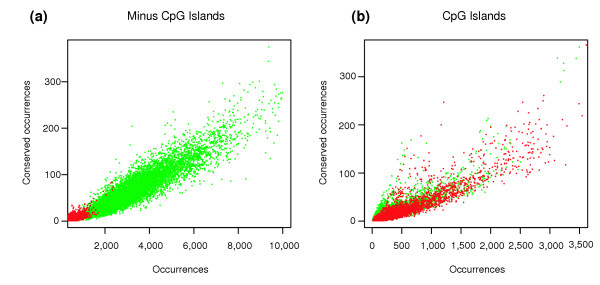
Two panels showing conservation versus occurrence of all 7-mer words upstream of genes split into **(a) **putatively unmethylated or **(b) **putatively methylated. In each case, only 5 kb upstream of genes was considered. Each word is colored either red if it has one or more CG dinucleotides or green otherwise.

We reasoned that dual behavior was unsurprisingly due to differential methylation of upstream regions giving rise to the well known signature of CpG islands. The problem is that we were combining two different types of regions (methylated versus unmethylated) with different word behaviors. There is no direct measurement of this methylation status genome-wide, so we used the classic observed versus expected ratio of CpG dinucleotides to make an approximate partitioning of our dataset. Importantly, we used far less stringent window lengths for valid sequences: we were not interested in predicted CpG islands in the context of the whole genome, but rather in predicting methylation status in the context of previously defined upstream regions. Figure 3 shows the now similar plots of the conserved versus total occurrence for these CpG (putatively unmethylated) and non-CpG (putatively methylated) regions. Now both distributions have the bulk of the CG containing motifs (red), behaving similarly to their non-CG containing peers (green), with the methylated regions showing the classic suppression of CG containing motifs. Interestingly, the CpG (putatively unmethylated) regions contain a larger quantity of significant points than the non-CpG (putatively methylated) regions, though both sets have significant motifs. These interesting motif points are both CpG containing motifs and non CG containing motifs and contain some purely AT motifs, in particular the classic TATA box (see below).

### Motif language enumeration

To perform a thorough search for motifs with significant objective function scores we used a suffix tree based method. This has the advantage that comparatively large pattern languages could be investigated quickly compared to simpler brute force enumeration strategies, such as using the standard regular expression in-built into many languages.

Pattern enumeration algorithms based on suffix trees have been previously published [20,33], but their use has been typically limited to prokaryotes and yeast because of their excessive memory requirements, despite requiring memory linearly in proportion to the total sequences used. Rather than use less memory demanding suffix arrays, here we have used an efficient but fast suffix tree memory scheme [34] to get the appropriate compromise between physical memory use and performance.

Choosing the appropriate pattern language was important for capturing as much useful information as possible. We tested pattern languages using both mismatches, where a specified number were allowed from the consensus sequence, and IUPAC ambiguity characters. Although both have merit, for many of our motifs with low information content, mismatches unrestricted in position could interrupt vital parts of the consensus sequence. We settled, therefore, on using a restricted subset of IUPAC ambiguity characters with motifs of between 5 and 12 bp long, where for speed of enumeration we excluded the triply redundant characters {BDHV}, and limited the total ambiguity of a consensus by a minimum information content.

Allowing degeneracy in motifs sets, however, poses a different challenge of deciding which precise motifs to report. Motifs can partially overlap each other (for example, TATAAT to AATGCGT have a three letter sequence in common), the partial overlap being even more prevalent when degenerate letters are allowed. In the process of enumeration, for each 'real' motif that is statistically significant, we expect many closely related motifs to also show significance. In addition, it is biologically feasible that partially overlapping motifs are more common than expected due to transcriptional control being mediated by either cooperativity or steric hindrance. We were inspired by the 'best explanator' approach of Blanchette [35] to solve the motif redundancy problem, but as the statistic has to be implemented in a space and time efficient manner, we developed a simpler approach along the lines of the same greedy approach (see Materials and methods).

The results of our scan for all 12-mers, allowing up to four positions to be fully redundant, found a total of 3.2 million unique motifs using the 'best explanator' method. At differing levels of degeneracy, subtly different collections of motifs were reported, and it is quite challenging to understand which of these motifs have been previously described. For annotation purposes, a scan with no degeneracy and applying the best explanator method resulted in 73 motifs in the CpG (unmethylated) set and 30 motifs in the non-CpG (methylated) set. In some cases this set still showed considerable degeneracy by eye, which we further manually merged. Table 1 lists these 55 motifs (some occur in both the CpG and the non-CpG sets), with any motif definition from the literature indicated. We found 12 of these 55 motifs in the Jaspar database. The only bias in these motifs is that they are generally the more 'basal' transcriptional motifs, present on many promoters. We found no bias in the length of the motif or occurrence in the genome, though most motifs occur in such vast excess of their expected functional number that such global occurrence ratings are unlikely to be meaningful. The results of our motif scans at a series of allowed degeneracy levels are listed in Additional data file 1, with the different degeneracy levels being potentially useful for different tasks. This list is clearly far short of the total number of expected motifs involved in transcription, which we expect due to the need for motifs to be involved in at least hundreds of promoter functions for them to show significance in our measure. We expand on this in the Discussion section.

Several known motifs are significant in our scan, in particular the CAAT box, SP-1site, and the TATA box (Table 1). The first two cases are examples where a number of similar motifs were found by the 'best explanator' method but where we believe there is only one core biological motif underlying these instances. This could indicate issues with the computational process of finding the best computational representation of a binding site or could be related to biological processes (for example, a particular subset of SP-1 sites that have a slight variation in structure). The fact that the TATA box also comes out in both the CpG and non-CpG cases is reassuring, and it is a good illustration of the power of this approach, as the motif itself is not over-represented in promoters and indeed is absent from a large number of promoters. We could not find evidence in the literature or in the Jaspar database for most of our sites, although it is extremely hard to find motif descriptions in the literature, and we apologize in advance for the cases that we have missed. The other novel motifs look in some cases like examples of sequence-specific binding sites, such as AAGATGGCGG, whereas a more degenerate motif such as TTTAAA is possibly not bound by a transcription factor but instead has a structural or some other role. There is no requirement, of course, that our motifs are actual binding sites, only that there are evolutionary advantages in keeping their base pair identity.

### Instance identification via distant comparative studies

The evolutionary selex approach provides us with a library of potential motifs, but does not specify which of the many instances of the motif in a genome is active. We first attempted to extend our comparative studies to more distant vertebrates (fugu, zebrafish, chicken and *Xenopus*). Even when controlling for the paucity of established 5' ends in other vertebrates, we observed that only a fraction of promoters (2% to 10%) had promoterwise alignments over 20 bits. We did not pursue using these high scoring alignments because of their low coverage, but we noticed that even in weak (below 20 bits) alignments between mammals and fish there were short word matches with our motifs. These low scoring alignments are ubiquitous and apparently indistinguishable from random alignments. Indeed, when we used a simple rule of scoring a motif as positive if we found a motif word match in the putative promoters to identify 43,052 specific instances of motifs in these genomes that matched at mammalian/fish distances. In many cases, the number of positive promoters having both a mammalian motif and a fish ortholog of a motif instance was clearly non-random, as judged by a hypergeometric probability of the co-occurrence. When we used randomized motif libraries or randomized ortholog sets, this signal was greatly reduced to between 2- and 10-fold less predictions per motif and, as expected, there were no significant hypergeometric motifs. As our original evolutionary selex predicted that the instances are enriched by at least five fold for real sites versus random sites, this additional screen means that the false discovery rate is between 1 in 10 and 1 in 100 depending on the motif. Clearly, this technique is limited by the lack of effective 5' end definition of genes in many of these species, but with this low false discovery rate this limitation mainly affects our sensitivity.

### Experimental validation

To directly assess the specificity of this approach we took advantage of the Medaka fish system, where transient transgenic experiments are usually consistently expressed over the eight days of development. We selected six instances from our comparative set at random from the specific instances on the *Fugu rubripes *genome, which acts as an effective surrogate for the Medaka genome. The respective promoter regions were cloned from the *Fugu rubripes *genome and inserted into a reporter vector. The reporter vector contains green fluorescent protein (GFP) as a reporter gene, which allows monitoring of expression *in vivo*. For an *in vivo *promoter assay, these constructs were tested by transient transgenesis using the I-SceI meganuclease protocol [36]. Embryos were screened 24 hours after injection (1 day post fertilization) for GFP expression. Five of the six promoters resulted in ubiquitous or specific expression in the time of analysis. For three of them (listed in Materials and methods), we generated both specific deletion constructs around the identified motifs and control deletions at a random location in the promoter. It proved difficult to generate the deletion constructs for the remaining two. Around 100 transgenic injections were done for each promoter and the expression patterns were scored in a double-blind manner (see Materials and methods).

**Figure 4:**
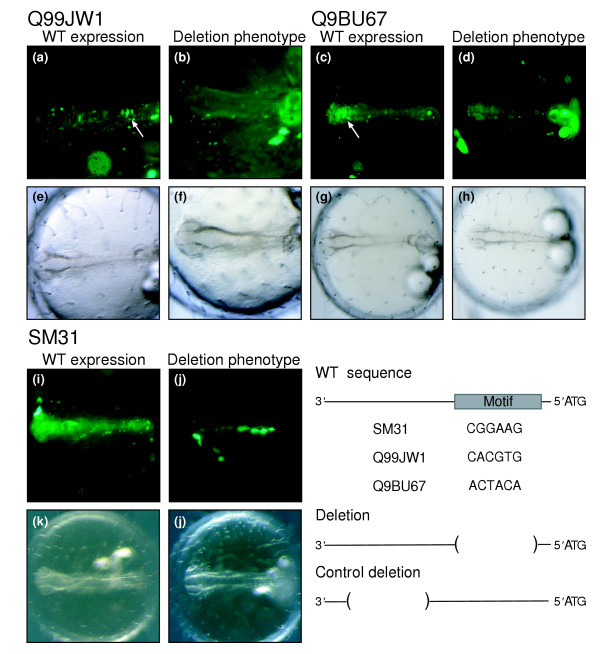
The three deletion mutants. **(a-d,i,j) **The predominant promoter activity for the indicated constructs, with **(e-h,k,l) **brightfield images as reference. (a) The arrow points to one of the strong clones, which is visible in many Q99JW1 wild-type (wt) fish compared to (b) the predominant deletion phenotype. (c) Similarly for the Q9BU67 native construct, the arrow in indicates an often found cluster of strong clones, which are hardly found in (d) the deletion construct injected fish. (i) SM31 shows ubiquitous expression found in most embryos injected with the native promoter, whereas (j) depicts the absence of green fluorescent protein expression with the deletion construct. These figures are representative examples from the large set of injections made for each construct (see Materials and methods). **(m) **Summary of the structure of the constructs used for reporter construction. Besides the native promoter, a construct was created with as precise a deletion of the motif as possible, together with a construct carrying a control deletion in a region presumably devoid of regulatory motifs.

**Figure 5:**
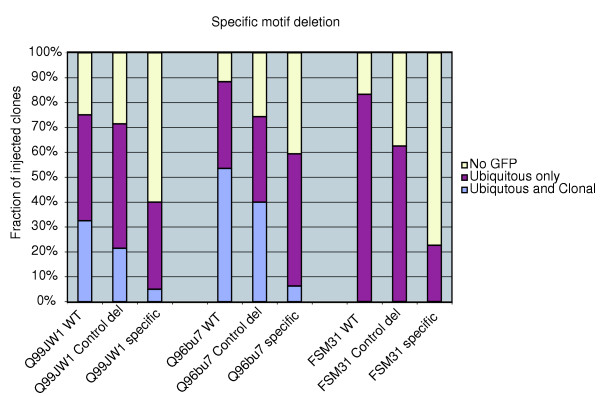
A bar chart summarizing 309 transgenic experiments. Each set of three bars represents a particular construct for a gene, labeled WT (wild type) for unaltered promoters, control del. for the control deletion of a random motif and specific for the specific deletion of the identified motif. In each bar, the proportions of embryos that were classified either as having no expression, ubiquitous only expression or ubiquitous with clonal expression are shown in yellow, maroon and blue, respectively. Each reporter was injected around 30 times and the expression patterns were scored in a double blind manner. GFP, green fluorescent protein.

All three promoters showed some ubiquitous expression and, for two of the genes (Q99JW1 and Q96BU7), there was often high GFP expression in specific clones of cells distributed along the entire embryonic axis (Figure 4a,b), indicative of cell type specific induction. This pattern of high expression in transient transgenic lines is a common feature of specific expression [36]. The specific deletion constructs showed both lower ubiquitous expression in all three cases, and in the case of Q99JW1 and Q96BU7, dramatically lower numbers of high expressing clones (for an example, see Figure 4). Figure 5 summarizes the results of 309 transgenic experiments and shows that there is a specific repression of both ubiquitous and the clonal GFP expression in the specific deletion compared to both wild-type (WT) and control deletion studies. The most striking case is Q96BU7 where clonal expression is present in 53% of the WT transgenics and 40% of the control deletions, but in only 6% of the specific deletion constructs. These results are clear evidence that these specific instances are involved in transcriptional control.

## Discussion

We have developed a new method, 'evolutionary selex', to find motifs involved in transcription using just genome sequence and transcript start sites, and have made significant specific predictions about which of these instances are actively controlling transcription. This method uses a highly specific set of negatively selected DNA, which we isolated using a novel alignment procedure. We show that this method finds many known motifs and several apparently novel cases. We have also shown by direct experiment that these motifs are involved in transcription.

The work of Xie *et al*. [28] shows similar results to ours for the first portion of our method. They use strict conservation across four mammals whereas we used a specific alignment routine between only the two most distant mammals in the set. In both cases, we discovered motifs by over-representation of motifs in conserved regions, with careful control of CpG effects. Our method only needs two genomes to be effective and, therefore, is useful for other clades for which fewer genomes are expected to be sequenced than for mammals. It is hard to compare lists of motifs directly because of the many arbitrary choices of where significant words start and end and the differing methods for reducing redundancy. Using a simple edit-distance measure, there is a large (67%) overlap between the two motif sets, suggesting both techniques are focusing on a similar class of motif. Another similarity of the two methods is the use of direct enumeration of words to find statistically interesting motifs; this is in contrast to model based approaches such as HMMs (Hidden Markov Meodels). Direct enumeration removes the need to be concerned with finding global optima, in contrast to local optima methods, and with suffix tree implementations it is not prohibitively costly in computational time.

The second part of this work, the prediction of specific instances of these motifs on the genome, is a significant advance beyond the work of Xie *et al*. [28]. Although they found many significant motifs, they are only able to show enrichment in conservation of these motifs and their bulk properties (for example, association with tissue expression patterns). Using more distant vertebrate sequences, we have overcome this limitation to make specific predictions of 43,052 motif instances. A surprise here is that, although the promoters of orthologous genes rarely have non-random alignments at even frog-human distances, word matching of specific motifs across vertebrates are both non-random and also provide experimentally verifiable predictions. There are two possible explanations for this behavior. Firstly, that we have the wrong alignment model for promoters, and in fact under the correct scoring scheme these motifs would be aligning. Secondly, that motif evolution involves *de novo *creation and destruction of these motifs over this timescale, and yet functional conservation of the presence of this motif in the promoter. We favor the latter explanation, but in either case this provides a very effective filter to find specific functional instances of motifs in the genome.

This specific instance identification has allowed us to test these computational predictions with specific experiments. This again is an advance over the Xie *et al*. method, which only provided correlation with previous expression datasets as indicative of their use in transcription. Here we show that these discovered motifs are involved in transcription in both general and cell type specific transcription. The ability to predict instances of active motifs is crucial to being able to design experiments for a particular gene, showing the utility of our instance discovery.

The map of motif instances across the genome is a first crude approximation of a genomic model of transcription. In particular, we have dramatically fewer motifs (55) than the estimated number of transcription factors in the human genome (at least 1,500 from protein domain content). This is not surprising, as our method is focused on ubiquitous factors, summing instances across multiple genes. In addition, this, like other approaches, has been a very promoter-proximal approach, also restricting the motifs to more basal factors. Over time, refinements of the negatively selected base pairs across mammals will help improve the statistical power of these methods. However, even this crude map will be useful for many downstream applications. For example, one might prioritize the analysis of single nucleotide polymorphisms that change these specific base pairs in disease searches, or use these motifs as a starting point for detailed experimental validation of a particular promoter. Clearly, both the construction of the motif dictionary and the mapping of specific instances must improve to provide a more detailed model for transcription. Overall, this is likely to require the integration of large scale experimental work, such as that being piloted in the ENCODE project [37], in combination with bioinformatics techniques.

## Materials and methods

### Promoterwise and genome searches

Promoterwise was written in the Wise2 package [38] using the dynamic programming macro language dynamite. The seeding system used an in-memory hash system that enumerated all 7-mers in one sequence (on both strands) as the other sequence was matched on each 7-mer, allowing for one mismatch by enumeration of all 3*6 one off differences. This was followed by a configurable step of extending motifs into high scoring segment pairs (HSPs) along a single diagonal and merging HSPs if they are close to each other (differing by under three gaps). The resulting local regions were extended by a default amount of 50 bp and then the DBA algorithm [30] was run on them. The resulting alignments were sorted by the log-odds bits score from the DBA algorithm, followed by a greedy procedure of accepting progressively lower scoring alignments only if they did not overlap previous alignments. In practice, for significant (>20 bits) alignments the alignments are nearly always disjoint.

All these programs are freely available under a GPL license in source code form at [38] as part of the Wise2 package.

### Sequence statistics for evolutionary selex

To assess the significance of motifs we needed to quantify the number of occurrences of a motif within a set of promoterwise alignments and background sequences from which the alignments were made. To do this we took only the human subsequences from the promoterwise alignments and the human set of background sequences. We avoided the issue of motif self overlap in counting by taking the total number of occurrences of a motif within a set of sequences as the maximum number of non-overlapping instances. To account for regional differences in the quality of alignment, we counted, after some experimentation, only completely conserved instances of a motif. Probabilities were calculated using the binomial distribution and converted into Z-scores. Clearly, this is an approximation to a more formal statistical model, but proved adequate for this genome-wide investigation.

To split the sequences into CpG enriched and negative regions we used the Emboss CpGIsland program with the default parameters but an adjusted minimum reportable length of 50 bp. To split the human alignment sequences we compared their overlap with the map of CpG islands and partitioned them accordingly.

### Motif discovery

To enumerate the binomial statistic for a given set of motif patterns we used the following algorithm. First we constructed a single suffix tree containing all our sequences using McCrieght's linear time algorithm [39] with the memory efficient scheme given in [34]. Practically, this meant we could hold a tree containing one hundred megabases of sequence in fewer than two gigabytes of working memory. In a similar fashion to the algorithm of Marsan and Sagot [34], we then traversed the tree enumerating patterns in an efficient lexicographic ordering, stopping where the number of occurrences fell below a threshold, and using multiple pointers where necessary to deal with degeneracy. The software was written in Java with the aims of speed and flexibility. In this scenario, we needed only to know total counts of motif occurrences within the two sets of sequence. In order, therefore, to prevent unnecessary continual re-enumeration of sub-trees we stored these counts at each node by a linear time preprocessing of the tree. Where a motif appeared promising, which we defined as having a preliminary Z-score of greater than ten, we reinvestigated the tree to resolve overlaps between instances of the motif.

To resolve the many high scoring variants of a motif into a single set of non-redundant motifs is a non-trivial problem. Adapting Blanchette's statistical approach to our method was desirable but considered both complex and too computationally expensive for the size of sequence we were using. We therefore adapted their simple greedy algorithm which, starting from the best scoring motif, filtered the sorted list according to compatibility with a growing set of non-degenerate motifs. Here we established compatibility by recalculating the Z-scores of motifs after counting the number of instances of a motif that did not overlap instances of motifs already in the non-degenerate set by greater than a third.

Annotation of our non-degenerate set of motifs was performed by a manual investigation of the literature assisted by an all against all alignment of our motif set to the Jaspar database of position weight matrices.

### Distant comparative studies

We scored each human promoter as positive for the motif if the motif was found in the context of a >20 bit promoterwise score for the human-mouse pair. We scored each fish promoter as positive if either the fugu or zebrafish promoter had an instance of the motif (irregardless of alignment). Zebrafish and fugu are a considerable evolutionary distance apart, so it is not feasible to perform the same alignment process as for human and mouse. A hypergeometric probability was calculated for the overlap of the mammalian and fish positive promoters using orthology links predicted by Ensembl, with the total number of fish/mammal orthologs as the background set (Ensembl version 18 was used throughout, but similar results were obtained with version 31).

### Molecular cloning

Genomic sequences that contained the conserved motif were retrieved from the Ensembl *F. rubripes *database. Upstream sequences of the respective genes of 1 to 2 kb in length were amplified by PCR. PCR primers were designed using Oligo 6.8 (Molecular Biology Insights, West Cascade, co USA) for fSM31, Q99JW1 and Q9BU67. Table 2 lists all the primers used. Upstream regions were amplified with proof reading Taq polymerase (Takara Bio Inc, Shiga, Japan) using 200 ng of *F. rubripes *genomic DNA as template. Cycling conditions were 35 cycles of 30 minutes at 94°C, 45 minutes at 59°C and 2 minutes at 72°C, followed by a final 5 minutes at 72°C on a Peltier Thermal cycler (PTC-2000, MJ research, Waltham, MA, USA).

PCR products of fSM31, Q99JW1 and Q9BU67 were cloned into pCRII-TOPO (Invitrogen, Carlsbad, CA, USA) and confirmed by sequencing before the insert was cloned into a pBlueScript-based transgenesis vector containing two recognition sites for the meganuclease ISce-I flanking a multiple cloning site and a 3' cassette containing enhanced GFP and a SV40-polyadenylation signal.

The deletion of the conserved motif in Q99JW1 was introduced by PCR using primers located 5' to the conserved motifs. For Q9BU67, fusion PCR was performed to precisely delete the 19 bp motif. The deletion in SM31 was introduced by restriction digest with *HinP1I *and *XhoI *(New England Biolabs, Beverly, MA, USA), cutting out a 62 bp fragment, and religation (T4 Ligase, Roche, Mannheim, Germany). All deletion constructs were sequenced.

### Injections

Injections were done as described [36]. Prior to injection, the DNA was purified using a spin column (nucleotide removal kit, Qiagen, Hilden, Germany). DNA was injected at concentrations of 15 ng/μl for fSM31 and Q99JW1 and 12 ng/μl for Q9BU67 using a Femtojet injector (Eppendorf, Hamburg, Germany).

### Screening

A Leica fluorescence dissection microscope (Leica MZ 16 FA, Leica Microsystems AG, Wetzlar, Germany) was used to examine GFP expression in live embryos. Injected embryos were analyzed for seven days to determine the spatial and temporal pattern of GFP expression. The elements fSM31 and Q99JW1 result in ubiquitous expression and embryos with a ubiquitous expression pattern were scored as positive. For Q9BU67, the intensity of the GFP fluorescence was included in the evaluation.

### Statistical analysis of expression data

Injections were repeated three times. Percentages of expression were weighted according to the total number of scored embryos for each injection. The statistical analysis was carried out using the Prism 4 software (GraphPad, San Diego, CA, USA) with a two-grouping variables table with three replicates. Mean and standard deviation were calculated using the standard algorithms in the program. A paired *t* test was carried out to determine the significance.

## Additional data files

The following additional data are available with the online version of this paper. Additional data file 1 is an Excel spreadsheet of the results of the motif finding method at different levels of degeneracy. The first sheet denotes positive motifs in CpG positive regions, the second sheet those in CpG negative regions. Each sheet contains three sets of two-column data. The first column indicates the motif, and the second column indicates the Z-score. Wild cards are represented as IUPAC ambiguity letters.

## Supplementary Material

Additional data file 1 The first sheet denotes positive motifs in CpG positive regions, the second sheet those in CpG negative regions. Each sheet contains three sets of two-column data. The first column indicates the motif, and the second column indicates the Z-score. Wild cards are represented as IUPAC ambiguity letters. Click here for file

## Figures and Tables

**Table 1:** Non-degenerate motifs found by the evolutionary selex (EvoSelex) method

Motif	Cluster size	Annotation	Reference	EvoSelex Z-score	Comparative *P *value
**CpG region motifs**					
CCAATC	11	CAAT-BOX	[40]	28.1	6.4e-6
GGGCGG	6	SP1	[41]	24.9	1.8e-8
TGACGTCA	3	CRE	[42]	23.8	2.8e-9
CGGAAG*	5	ETS	[43]	23.4	3.6e-9
CACGTG*	1	E-Box	[44]	23.1	3.3e-7
ACTACA*	3			20.4	6.0e-5
GTGACG	2	CRE related	[42]	16.5	5.0e-4
CTTTGT	2			16.1	0.5
CCCTCCCCC	5	SP1 related	[41]	15.9	0.05
GCGCAGGCGC	2			15.5	1.0e-3
GCGCGC	1			15.5	4.6e-13
AACTTT	4			15.4	0.3
CCTTTAA	3			15.3	0.01
TGCGCA	1			14.6	2.7e-5
CTCGCGAGA	1			14.6	4.13e-8
TTGGCT	1			13.9	0.01
TATAAA	1	TATA-box	[45]	13.7	0.49
AAGATGGCGG	1			13.6	0.001
TTTGTT	3			13.4	0.13
ATGCAAAT	1			13.3	1.0e-4
TAATTA	1			13.1	0.06
TTTAAG	1			13.1	0.5
CGCATGCG	1			13.1	1.1e-5
ATAAAT	1			12.6	0.02
TTTAAA	1			12.6	0.02
GCCATTTT	1			11.7	8.5e-7
ATAAAA	1			11.7	0.6
TAAATA	1			11.6	0.5
CAGGTG	1	Helix-turn-helix	[46]	11.2	0.2
CTAGCAAC	1			11.0	4.0e-3
TGACGC	1	CRE	[42]	10.9	1.6e-4
CATTGT	1			10.7	0.14
GCCATCTT	1			10.6	8.4e-5
ATTTAT	1			10.6	0.02
ATGAAT	1			10.2	9.0e-3
					
**Non-CpG region motifs**					
TAATTA	1			20.3	0.064
CAGCTG	1			18.4	0.31
TGAGTCA	2	TRE	[47]	18.1	8.0e-3
CAGGAAGT	5	ETS	[43]	14.9	0.79
CCCTCCC	2			14.4	2.68e-10
AATAAA	2			14.0	0.31
AATTAA	2	Homeodomain related	[48]	13.5	0.17
AGAAAA	2			12.9	0.44
ATAAAA	1			12.7	0.68
TTTCCA	2			12.5	0.04
TATAAATAG	1	TATA-box	[45]	12.2	0.01
AGGAAA	1			12.2	0.091
TTTCCT	1			12.2	0.091
TTCAAA	1			12.1	0.079
TGACCT	1			11.7	0.040
ATTTGCAT	1			11.3	1.0e-4
TTGTTT	1			10.9	0.011
TTTAAA	2			10.7	0.020
TTTCAG	1			10.4	0.31

The first column gives the motif consensus. *The three tested motifs in the experimental validation. The second column gives the number of related motifs when by hand analysis was used to remove additional redundancy. The third column gives a brief text description when we found a matched motif, and the literature reference for these cases is shown in the fourth column. The fifth column gives the Z-score (the number of standard deviations from the expected mean) for the conserved versus occurrence ratio on the basis of the binomial distribution. The sixth column is the probability of observing the overlap between fish and human promoters containing this motif. The table is sorted by Z-score.

**Table 2:** PCR primers used

Upstream region	Construct	Direction	Sequence
SM31	Control	Sense	5'-CCTTCAGGAGCCTCAACAACAACAAAT-3'
		Antisense	5'-ACAAATGAATGATTGGTCCCGACACGA-3'
Q99JW1	Control	Sense	5'-GCGCTCCTCTCCCGTTATTGTTCAGGC-3'
		Antisense	5'-CCTCTGTCCATCCATGCTACTGACCGA-3'
	Deletion	Antisense	5'-CGTCACGGGCGCTTCCATTTCAAAACT-3'
Q9BU67	Control	Sense	5'-CTCTGTCAAGAAAGTGATGCCGTGAAA-3'
		Antisense	5'-GAATGTTCAGAAGAGCAGCCGAGGGAT-3'
	Deletion	Sense	5'-CCAGGCCACGTTGTTATTTTGCTTCCGC-3'
		Antisense	5'-AAAATAACAACGTGGCCTGGCCAGAGCC-3'
